# Whole Genome Sequencing Indicates Heterogeneity of Hyperostotic Disorders in Dogs

**DOI:** 10.3390/genes11020163

**Published:** 2020-02-04

**Authors:** Anna Letko, Fabienne Leuthard, Vidhya Jagannathan, Daniele Corlazzoli, Kaspar Matiasek, Daniela Schweizer, Marjo K. Hytönen, Hannes Lohi, Tosso Leeb, Cord Drögemüller

**Affiliations:** 1 Institute of Genetics, Vetsuisse Faculty, University of Bern, 3012 Bern, Switzerland; anna.letko@vetsuisse.unibe.ch (A.L.); fabileuthard@gmail.com (F.L.); vidhya.jagannathan@vetsuisse.unibe.ch (V.J.); tosso.leeb@vetsuisse.unibe.ch (T.L.); 2 Clinica Veterinaria Roma Sud, 00173 Roma, Italy; daniele.corlazzoli@me.com; 3 Section of Clinical & Comparative Neuropathology, Centre for Clinical Veterinary Medicine, Ludwig Maximilians Universität Munich, 80539 Munich, Germany; matiasek@patho.vetmed.uni-muenchen.de; 4 Division of Clinical Radiology, Vetsuisse Faculty, University of Bern, 3012 Bern, Switzerland; daniela.schweizer@vetsuisse.unibe.ch; 5 Department of Medical and Clinical Genetics, and Department of Veterinary Biosciences, University of Helsinki, 00014 Helsinki, Finland; marjo.hytonen@helsinki.fi (M.K.H.); hannes.lohi@helsinki.fi (H.L.); 6 Folkhälsan Research Center, 00290 Helsinki, Finland

**Keywords:** whole-genome sequencing, craniomandibular osteopathy, calvarial hyperostotic syndrome, Caffey disease, infantile cortical hyperostosis, rare disease, *SLC37A2*, *COL1A1*, *SLC35D1*

## Abstract

Craniomandibular osteopathy (CMO) and calvarial hyperostotic syndrome (CHS) are proliferative, non-neoplastic disorders affecting the skull bones in young dogs. Different forms of these hyperostotic disorders have been described in many dog breeds. However, an incompletely dominant causative variant for CMO affecting splicing of *SLC37A2* has been reported so far only in three Terrier breeds. The purpose of this study was to identify further possible causative genetic variants associated with CHS in an American Staffordshire Terrier, as well as CMO in seven affected dogs of different breeds. We investigated their whole-genome sequences (WGS) and filtered variants using 584 unrelated genomes, which revealed no variants shared across all affected dogs. However, filtering for private variants of each case separately yielded plausible dominantly inherited candidate variants in three of the eight cases. In an Australian Terrier, a heterozygous missense variant in the *COL1A1* gene (c.1786G>A; p.(Val596Ile)) was discovered. A pathogenic missense variant in *COL1A1* was previously reported in humans with infantile cortical hyperostosis, or Caffey disease, resembling canine CMO. Furthermore, in a Basset Hound, a heterozygous most likely pathogenic splice site variant was found in *SLC37A2* (c.1446+1G>A), predicted to lead to exon skipping as shown before in *SLC37A2*-associated canine CMO of Terriers. Lastly, in a Weimaraner, a heterozygous frameshift variant in *SLC35D1* (c.1021_1024delTCAG; p.(Ser341ArgfsTer22)) might cause CMO due to the critical role of *SLC35D1* in chondrogenesis and skeletal development. Our study indicates allelic and locus heterogeneity for canine CMO and illustrates the current possibilities and limitations of WGS-based precision medicine in dogs.

## 1. Introduction

Craniomandibular osteopathy (CMO) in dogs is a common type of a hyperostotic disorder, which mainly affects the mandible (OMIA 000236-9615). CMO is a developmental orthopedic disorder described in several dog breeds, and it is clinically equivalent to human infantile cortical hyperostosis, also known as Caffey disease [1] (OMIM 114000). The clinical signs in human patients appear under 6 months of age and are usually self-regressive. This includes swelling and inflammation of soft tissues, as well as hyperostosis of the facial bones, especially the mandible [2]. The disease typically follows an autosomal dominant mode of inheritance with reduced penetrance and has been associated with a common missense variant (NP_000079.2: p.Arg1014Cys) in the *COL1A1* gene [3]. However, genetic heterogeneity exists because this variant was not observed in some of the human Caffey-affected patients [3,4,5]. Furthermore, an autosomal recessive type of Caffey disease was recently described, reporting a nonsense variant in the *AHSG* gene that encodes alpha 2-HS glycoprotein involved in the formation of bone tissue [6].

CMO in dogs was first described in 1958 [7], and since then it has been observed in several dog breeds including Labrador Retriever, Doberman Pinscher, Great Dane, Boxer, English Bulldog, West Highland White Terrier, Shetland Sheepdog, Pyrenean Mountain Dog, Bullmastiff, Akita, Pit Bull Terrier, Airedale Terrier, Scottish Terrier, Cairn Terrier, and German Wirehaired Pointer (OMIA 000236-9615). This proliferative bone disorder is usually seen in young, growing dogs up to 12 months of age and affects both sexes equally. Clinically it is characterized by a painful swelling of the jaw and the consequent discomfort signs (e.g., difficulty in opening mouth, salivation, and dysphagia that might result in malnutrition) [8]. In addition, the inflammation of soft tissues often results in fever during the bone proliferation phase. The hyperostotic disorder is considered self-limiting, and all clinical signs usually resolve with time, when the regular bone growth and ossification are complete [7]. Calvarial hyperostotic syndrome (CHS) also belonging to the group of canine hyperostotic disorders clinically resembles canine craniomandibular osteopathy and is not histopathologically distinguishable from CMO. Both CHS and CMO may be associated with changes of the long bones, consistent with hypertrophic osteodystrophy. On the basis of histology, it has been proposed that CMO, calvarial hyperostosis, and hypertrophic osteodystrophy belong all to the same osteoproliferative or hyperostotic disorder complex depending on the bones affected [9]. 

Different modes of inheritance have been reported for canine CMO, suggesting heterogeneity and distinct breed-specific variants. Padgett and Mostosky [10] showed evidence for a recessive mode of inheritance in the West Highland White Terriers by retrospective pedigree analysis. In a genome-wide association study, a splicing variant in the *SLC37A2* gene (XM_005619600.3:c.1332C>T) was identified as causative in West Highland White Terriers, Cairn Terriers, and Scottish Terriers. CMO in these Terrier breeds is inherited with a dominant mode of inheritance with incomplete penetrance [11]. Recently, a more complex polygenic inheritance for CMO in German Wirehaired Pointers was suggested using statistical segregation analysis [12]. Therefore, the understanding of the underlying genetics of canine hyperostotic disorders might benefit from a precision medicine approach.

Germline genetic variation is a quite frequent causal factor in rare diseases in humans. Whole-genome sequencing (WGS) is a relatively inexpensive method to determine individual variation [13]. Precision medicine by using WGS has been proven suitable in recent years to help diagnose rare diseases in single patients and situations with limited access to patients [14]. The methodology has also been successfully adapted to veterinary medicine. For example, single dogs showing suspicious inherited disorders were sequenced and compared to hundreds of normal controls [15], which allowed a precise molecular diagnosis reporting the most likely disease-causing variants [16,17,18]. A similar approach has also been successfully applied to cats [19,20,21]. By applying short-read WGS as the current gold standard in precision medicine, we aimed to identify further plausible variants associated with canine hyperostotic disorders such as CMO and CHS to improve the understanding of the etiology of these rare genetic disorders.

## 2. Materials and Methods 

### 2.1. Ethics Statement

All animal experiments were performed according to the local regulations. All animals in this study were examined with the consent of their owners. Sample collection was approved by the Cantonal Committee for Animal Experiments (Canton of Bern; permit 75/16).

### 2.2. Animals

Blood samples from a CHS-affected American Staffordshire Terrier and seven dogs affected by CMO of seven different breeds (Supplementary Table S1) were collected. Genomic DNA was isolated from EDTA blood samples using the Maxwell RSC Whole Blood DNA kit (Promega, Dübendorf, Switzerland). All dogs were diagnosed by respective veterinarians on the basis of clinical signs and imaging. Skull radiographs of four and CT scan images of two of the CMO-affected dogs were obtained.

### 2.3. Whole-Genome Resequencing

WGS data of the eight affected dogs were obtained after preparation of a PCR (polymerase chain reaction)-free fragment library at an average 26× coverage. Fastq-files were mapped to the dog reference genome assembly CanFam3.1. Single nucleotide and small indel variants were called and NCBI (National Center for Biotechnology Information) annotation release 105 was used to predict their functional effects, as described previously [15]. Private variants were identified by comparison with the variant catalog of 576 publically available dogs of 120 various breeds, as well as 8 wolves (Supplementary Table S1), provided by the Dog Biomedical Variant Database Consortium (DBVDC) [15]. The description of the sequence variants is in accordance with the recommendations of the Human Genome Variation Society [22]. Integrative genomics viewer (IGV) [23] was used for visual inspection and validation of the candidate variants found in the affected dogs’ WGS data.

### 2.4. Protein and Splice Site Predictions

The Genome Aggregation Database (gnomAD) [24] was searched for corresponding variants in the human *COL1A1*, *SLC37A2*, and *SLC35D1* genes. PredictSNP [25] and PROVEAN [26] were used to predict the biological consequences of the discovered *COL1A1* variant on the protein. SpliceRack tools [27] were used to evaluate the *SLC37A2* splice site variant. All references to the canine *COL1A1*, *SLC37A2*, and *SLC35D1* genes correspond to the accessions NC_006591.3 (chromosome 9) and NC_006587.3 (chromosome 5) (NCBI accession); NM_001003090.1, XM_005619600.3, and XM_003434643.2 (mRNA); and NP_001003090.1, XP_005619657.1, and XP_003434691.1 (protein), respectively. The canine COL1A1 protein has 1460 amino acids compared to the human protein (NP_000079.2) with 1464 amino acids, from which 98% are identical between dog and human. The canine SLC37A2 protein has 508 amino acids compared to the human protein (NP_001138762.1) with 501 amino acids, from which 86% are identical between dog and human. The canine SLC35D1 protein has the same length of 355 amino acids compared to the human protein (NP_055954.1), from which 98% are identical between dog and human. For the multi-species COL1A1 protein alignment, the following NCBI accession numbers for each species were used: NP_001003090.1 (*Canis familiaris*), NP_000079.2 (*Homo sapiens*), NP_001029211.1 (*Bos taurus*), NP_031768.2 (*Mus musculus*), NP_445756.1 (*Rattus norvegicus*), NP_001011005.1 (*Xenopus tropicalis*), and NP_954684.1 (*Danio rerio*).

### 2.5. Availability of Data and Material

The WGS data of all affected as well as comparison cohort dogs/wolves have been made freely available at the European Nucleotide Archive (ENA). All ENA accession numbers are given in the Supplementary Table S1.

## 3. Results

### 3.1. Phenotype

**Figure 1:**
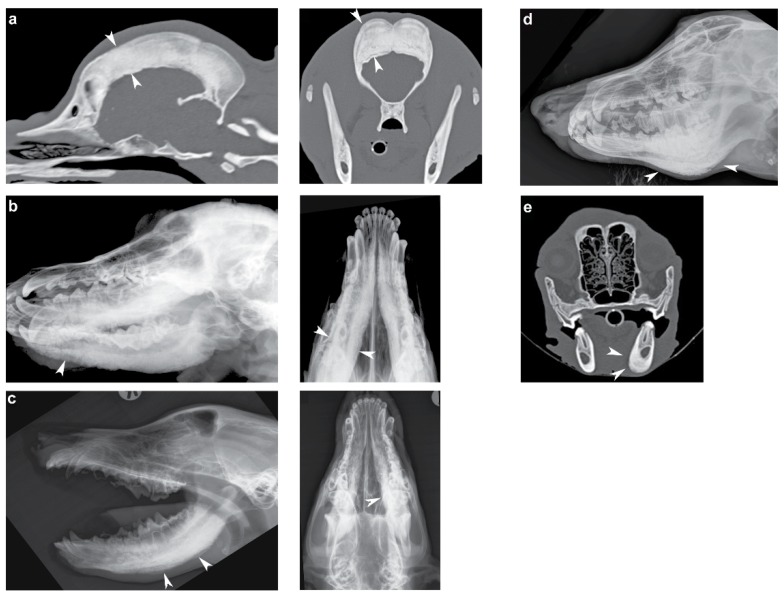
Radiographs/computed tomographic (CT) images of the craniomandibular osteopathy (CMO)/calvarial hyperostotic syndrome (CHS)-affected dogs. (**a**) Sagittal and transverse CT images of an American Staffordshire Terrier showing severe thickening of the calvarial diploic bone (arrowheads). (**b**) Ventrolateral-dorsolateral oblique and ventrodorsal skull radiographs illustrating a palisading periosteal new bone formation along the mandible of a Basset Hound (arrowheads). (**c**) Open mouth laterolateral and ventrodorsal view of the skull of a German Wirehaired Pointer showing periosteal new bone formation along the mandible (arrowheads). (**d**) Slightly oblique laterolateral view of the mandible of an Old English Sheepdog showing similar periosteal new bone formation along the mandible (arrowheads). (**e**) Transverse CT image of a Weimeraner with the arrowheads pointing towards the periosteal new bone formation at the ventral and medial aspect of the left mandibular corpus.

We investigated a CHS-affected American Staffordshire Terrier and seven CMO-affected dogs of different breeds (Australian Terrier, Basset Hound, Cairn Terrier, Curly Coated Retriever, German Wirehaired Pointer, Old English Sheepdog, and Weimaraner). The diagnosis was made by veterinarians observing the typical clinical signs including persistent and sharp pain when opening mouth, painful swelling of the jaw, fever, and in some cases also severe pain of ulna and radius bones. Age of onset of the clinical signs ranged from 3 to 7 months of age, and skull radiographs/computed tomographic (CT) images were available for six of the affected dogs (Supplementary Table S1). The imaging findings of one dog was consistent with CHS and in four dogs with CMO (Figure 1), in the remaining dog, no changes were identified on radiographs, but clinical signs were consistent with the diagnosis of CMO. Due to the self-regressive course of the disorder, the remission of clinical signs appeared in all cases soon after the diagnosis and symptomatic treatment. On the basis of the previously published gene test, all eight collected dogs were genotyped negative for the reported CMO-causing variant in *SLC37A2* (XM_005619600.3:c.1332C>T) [11]. In addition, for all eight dogs, the region of the *SLC37A2* gene was also manually inspected in IGV to confirm no candidate variants were missed in the functional annotation and to rule out obvious, large structural variants including copy number variation affecting this gene.

### 3.2. Identification of Private Candidate Variants

**Figure 2:**
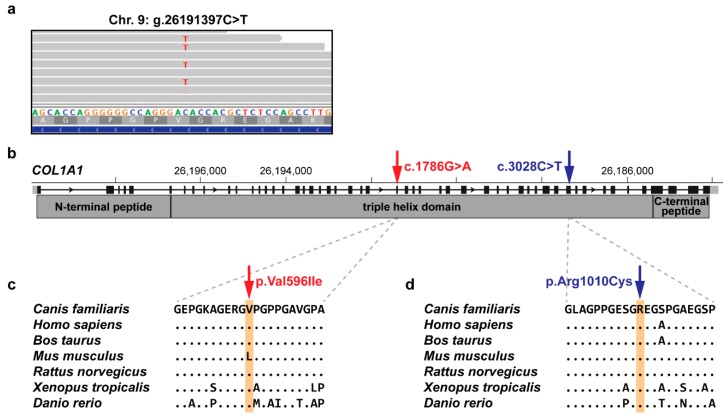
Characterization of the identified functional candidate variant in *COL1A1* in the CMO-affected Australian Terrier. (**a**) Integrative genomics viewer (IGV) snapshot showing the heterozygous missense candidate variant (NC_006591.3:g.26191397C>T). (**b**) Schematic representation of *COL1A1* indicating the c.1786G>A variant location in exon 26 (red) and the previously described human variant (position with respect to the canine *COL1A1*) in exon 41 (blue) encoding the triple helix domain. Note that the canine *COL1A1* gene is annotated on the reverse complementary strand. (**c**) Multispecies protein alignment of the canine CMO-associated missense variant identified herein (red). (**d**) Multispecies protein alignment of the human disease-causing missense variant (blue) [3] with respect to the canine COL1A1 position.

**Figure 3:**
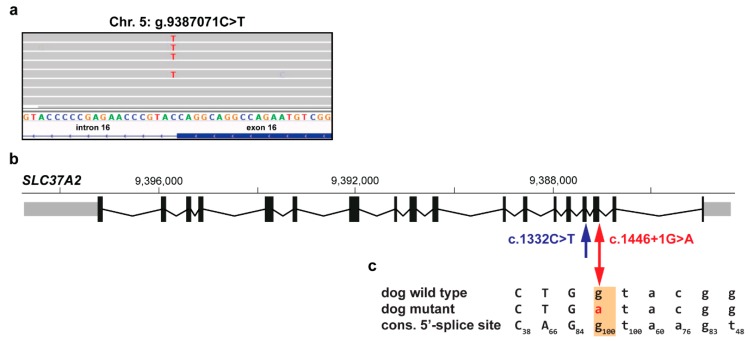
Characterization of the most likely pathogenic variant in *SLC37A2* in the CMO-affected Basset Hound. (**a**) IGV snapshot showing the heterozygous splice site candidate variant (NC_006587.3:g.9387071C>T). (**b**) Schematic representation of the *SLC37A2* transcript (XM_005619600.3) including the location of previously identified canine splicing variant at the end of exon 15 [11] (blue), as well as the newly reported variant at the beginning of intron 16 (red). Note that the *SLC37A2* gene is annotated on the reverse complementary strand. (**c**) Wild type and mutant allele compared to the human consensus sequence for the U2-type GT-AG donor splice sites [27]. The uppercase letters indicate exon 16 and lowercase letters intron 16 sequence, whereas the subscript numbers show the percentage of the respective conserved nucleotide in 186,630 investigated U2-type GT-AG human 5’-splice site motifs. The base at position +1 is a highly conserved G (100%) [27].

**Figure 4:**
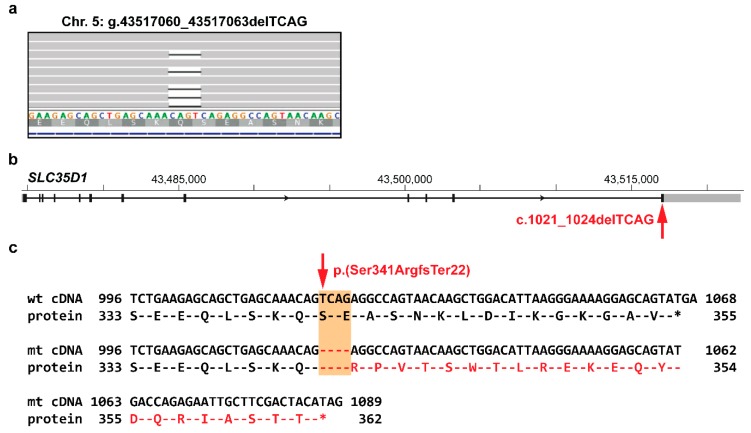
Characterization of the identified functional candidate variant in *SLC35D1* in the CMO-affected Weimaraner. (**a**) IGV snapshot showing the heterozygous frameshift candidate variant (NC_006587.3:g.43517060_435178063delTCAG), note that the variant description is adapted to the 3’ rule [22]. (**b**) Schematic representation of the *SLC35D1* gene with the variant affecting the last exon. (**c**) Comparison of wild type and the predicted mutant C-terminus protein sequence based on cDNA entry XM_003434643.2. The variant (XP_003434691.1:p.(Ser341ArgfsTer22)) affects the last 15 amino acids of SLC35D1.

We considered both the autosomal dominant and autosomal recessive modes of inheritance when analyzing the WGS data. Filtering for private variants present in a homozygous or heterozygous state in all eight affected dogs and absent in 584 publicly available dog and wolf genomes revealed no variants shared across all cases. However, filtering for private variants of each of the cases separately yielded possible dominantly inherited candidate protein-changing variants in three cases (Figure 2, Figure 3 and Figure 4). In the remaining five affected dogs, no variants in obvious functional candidate genes were found. Therefore, we report a catalog of each dog’s private variants (Supplementary Tables S2–S9). The total number of variants per genome, when compared to the CanFam3.1 reference, ranged from 4.9 million in the American Staffordshire Terrier to 5.6 million in the Basset Hound (Table 1). After filtering against the public genomes, on average ≈9200 variants were left with the least count observed in American Staffordshire Terrier (4582 variants) and the highest in the Basset Hound (13,370 variants), as shown in Table 1. In total, most of the called variants were located in non-coding intergenic (50.7%) or intronic (48.3%) regions, whereas on average 64.5 (34–96) protein-changing (0.7%) variants were found in each genome.

In the CMO-affected Australian Terrier, a missense variant in exon 26 of the alpha 1 chain of type I collagen (*COL1A1*) gene was discovered. The detected variant (NM_001003090.1:c.1786G>A) alters the encoded amino acid residue 596 of COL1A1 (NP_001003090.1:p.(Val596Ile)) with a predicted moderate impact on the resulting protein (Figure 2). The missense variant affects the triple helix domain of COL1A1 protein, which typically consists of the invariant Gly-X-Y repeat motif. The amino acid is conserved across species but the valine to isoleucine substitution was predicted neutral and tolerated by in silico prediction tools [25,26]. In humans, a similar missense variant has been described in exon 41, also changing the X residue in the triple repeat motif (Figure 2). There is no non-synonymous variant in the human *COL1A1* coding region reported at the corresponding position in the gnomAD [24].

In the CMO-affected Basset Hound, a splice site variant was found in the solute carrier family 37 member 2 (*SLC37A2*) gene. This single nucleotide variant (XM_005619600.3:c.1446+1G>A) affects an evolutionary strongly conserved donor splice site at the beginning of intron 16 and was predicted to disrupt splicing (Figure 3). The human consensus sequence for the U2-type GT-AG donor splice site corresponds to a perfect base pairing to the 5′ end of U1 small nuclear RNA [27]. The variant changes this canonical dinucleotide sequence (Figure 3c) and therefore most likely eliminates the splice site and leads to skipping of exon 16 and a shortened transcript. One human variant (rs1278346722) alters the same position of the human *SLC37A2* donor splice site as the dog variant; however, the guanine is exchanged by cytosine (c.1425+1G>C), not by adenine as found here. This human variant is present in only 2 out of 125,127 patients in a heterozygous state (allele frequency of 7.99 × 10^−6^) [24].

Lastly, a frameshift variant in the solute carrier family 35 member D1 (*SLC35D1*) gene (XM_003434643.2:c.1021_1024delTCAG) was found in a CMO-affected Weimaraner. The schematic representation indicates that the 4 bp deletion is located in exon 12 and leads to a frameshift affecting the last 15 amino acids of the SLC35D1 protein, and was predicted to produce a novel 22 amino acid long C-terminus of SLC35D1 (XP_003434691.1:p.(Ser341ArgfsTer22); Figure 4). There is no non-synonymous variant in the human *SLC35D1* coding region at the corresponding position described in the gnomAD [24].

## 4. Discussion

On the basis of clinical signs and/or radiography analyses, eight dogs of eight different breeds were diagnosed with different forms of hyperostotic disorders such as CHS or CMO. Because all eight affected dogs were homozygous wild type for the previously described variant causing CMO in three different Terrier breeds [11], whole-genome sequencing of the individual cases was performed to investigate the underlying genetics.

No variants shared across all 8 affected dogs and absent from 584 publically available genomes were discovered, which was in accordance with the expectation of several breed-specific types. The total number of private variants varied greatly between the individual genomes. This might be partially the consequence of different levels of inbreeding in different breeds, as well as the variable numbers of breed controls present in the studied dataset [15]. By prioritization of functional candidate genes, three possible causative variants in *COL1A1, SCL37A2,* and SLC35D1 were identified when searching for private variants in the Australian Terrier, the Basset Hound, and the Weimaraner, respectively.

The identified *COL1A1*:c.1786G>A variant in a CMO-affected Australian Terrier was predicted to result in a substitution of valine by isoleucine in the triple helix domain of alpha 1 chain of type I collagen (*COL1A1*). The structure of connective tissues includes collagens, proteoglycans, and non-collagenous proteins, as well as enzymes that are necessary for precise extracellular matrix assembly and degradation. Mutations in genes encoding these distinct components of the matrix result in similar phenotypes [28]. The triple-helical domain in collagen proteins consists of a Gly-X-Y repeating motif that is essential for the helix folding [29]. Mutations of the Gly residues lead to more severe defects such as osteogenesis imperfecta (OMIM 120150, OMIA 002126-9615, OMIA 002127-9913). On the other hand, substitutions of the X or Y residues result in milder phenotypes and are associated with Ehlers–Danlos syndrome and Caffey disease (OMIM 120150) [28]. The autosomal dominant form of infantile cortical hyperostosis in humans (OMIM 114000) is described as a collagenopathy caused by a single heterozygous missense variant in *COL1A1* with incomplete penetrance. This variant affects the X residue in a triple-helical domain of COL1A1 (Figure 2), but the exact pathogenesis of how it leads to self-limiting hyperostotic bone lesions is still unclear [3]. Therefore, we speculate that the identified missense variant in canine *COL1A1* might represent a candidate variant of uncertain significance for CMO in the affected Australian Terrier. However, as the predicted amino acid substitution involves two chemically similar, hydrophobic amino acids, this result should be considered preliminary and requires further confirmation.

The detected splice site variant in *SLC37A2* is the most likely pathogenic variant for CMO in the affected Basset Hound. *SLC37A2* has been shown to be associated with CMO in Terriers [11]. Belonging to the glucose-phosphate transporter family, it is expressed in bone-related tissues including bone marrow or osteoclasts. In mice, *Slc37a2* was shown to have an important role in osteoclast differentiation and function [30]. The previously identified synonymous variant causes altered splicing, resulting in a frameshift and premature stop codon [11]. This likely affects the function of SLC37A2 and leads to disturbed glucose homeostasis in osteoclasts in the developing bones, potentially explaining the hyperostosis phenotype of CMO-affected Terriers [11]. The process of splicing consists of the recognition of exon-intron boundaries by spliceosome complex and the subsequent excision of an intron and is highly conserved across eukaryotes [31]. Splice sites are classified in four major subtypes on the basis of components of the spliceosome and the introns’ terminal dinucleotides. The majority of all human splice sites are the U2-type GT-AG subtype. The highly conserved bases at the first and second positions of the 5’ motif of U2-type splice sites are G and T (100%) [27]. The herein reported splice site variant (c.1446+1G>A) detected in a CMO-affected Basset Hound changes this canonical dinucleotide sequence. Therefore, it most likely disrupts the correct splicing of intron 16.

The *SLC35D1* protein-changing variant (c.1021_1024delTCAG) found in a CMO-affected Weimaraner leads to a frameshift affecting the last 15 amino acids of the SLC35D1 protein. The solute carrier family 35 member D1 (*SLC35D1*) gene encodes another nucleotide-sugar transporter, which has a crucial role in chondroitin sulfate biosynthesis [32]. Chondroitin sulfate chains are an important part of cartilage proteoglycans, which are needed for chondrogenesis and skeletal development [33]. Impairment of the proteoglycan synthesis was confirmed in the *Slc35d1*-knockout mice, which showed severe chondrodysplasia, including hypoplastic craniofacial bones and extremely short long bones. The heterozygous *Slc35d1^+/-^* mice were phenotypically apparently normal [32]. In humans, autosomal recessive mutations in *SLC35D1* have been described in a rare lethal skeletal dysplasia (OMIM 269250). On the basis of these comparative data, it seems possible that the canine frameshift variant represents a likely causative variant for CMO phenotype in the affected Weimaraner. This might be either due to haploinsufficiency or a dominant negative function of the mutant protein on the residual wildtype SLC35D1.

The success rate of WGS-based identification of rare disease-causing genes is difficult to determine because about two-thirds of studied human diseases discover too many credible candidate variants and in the remaining one-third there are none [13]. Similarly, the comparisons described herein, using the recently presented DBVDC variant catalog, reduced the ≈5,000,000 variants per genome identified from WGS to <100 protein-changing variants per genome. Even though the DBVDC variant catalog is a great resource for identification of potential causal variants in dogs, the numbers of identified private protein-changing variants per genome are probably overestimated because of the still imperfect status and annotation of the current CanFam3.1 reference genome, and prioritization of causal variants remains challenging. For example, there is a gap in the reference assembly in the 5’-region of *SLC37A2*. Therefore, sequencing of additional genomes of well-phenotyped breed-matched obligate carriers and/or affected dogs may help unravel the complete molecular etiology of canine hyperostotic disorders such as CHS or CMO in the remaining cases by reducing the number of private candidate variants shared in cases of the same breed. De novo mutations causing autosomal dominant disorders are easier to identify when parents’ data is available because each individual carries fewer variants that are not also found in the parents [16]. Unfortunately, for the examined cases in this study, we had no access to samples of the parents to evaluate the possibility of spontaneously occurring mutations causing the hyperostotic disorder. Furthermore, analyzing the transcriptome of biopsies or even better relevant cells from affected tissue by RNAseq methods would be an excellent option to get further insights into the development of this transient disease phenotype. Despite the availability of the molecular methods, studying possible temporal and/or cell-specific expression changes remains challenging due to the practical and ethical limitations of accessing the appropriate samples in privately-owned dogs.

Considering the relatively mild and self-limiting phenotype, some cases may remain undiagnosed, which together with lower penetrance possibly prevented the identification of further disease-causing variants. Comparison cohort dogs used for variant filtering were assumed to be non-affected. However, in light of the fact that the affected individuals with CHS or CMO spontaneously recover, we cannot rule out the possibility that pathogenic alleles were present in the publically available genomes, which would obviously limit the power of the WGS-based precision medicine approach.

## 5. Conclusions

We propose that the novel splicing variant *SLC37A2*:c.1446+1G>A is most likely pathogenic for CMO. We report two other candidate causative variants of uncertain significance with less evidence for pathogenicity in compelling functional candidate genes (*COL1A1* and *SLC35D1*). Further functional studies are needed to confirm the causality of the described variants, whereas additional genetic and/or environmental factors might also influence the manifestation of the disorder. In addition, the detection of structural variants such as larger-sized indels or segmental duplications is strongly limited. The sensitivity of our variant detection might have been compromised by the presence of gaps in the reference genome and the employed short-read sequencing technology. Consequently, long-read sequencing improved reference assembly and annotation quality, and better algorithms for indels and structural variant calling might overcome these issues in the future. Nevertheless, our study demonstrates allelic and locus heterogeneity of different forms of canine hyperostotic disorders and indicates the current possibilities and limitations of precision medicine in dogs.

## Figures and Tables

**Table 1:** Private variants detected in the individual genomes of the 8 affected dogs and absent in the 584 comparison cohort genomes. The table gives total counts of private variants divided into classes on the basis of their predicted effect. The counts of homozygous/heterozygous genotypes separately are shown in parentheses.

Affected Dog	Total Variants in the Whole Genome	Private Variants after Filtering against 584 Publically Available Genomes				
		Total	Protein-Changing	Synonymous	Intronic	Intergenic
American Staffordshire Terrier (CHS)	4,861,208	4582	76 (67/9)	71 (69/2)	2217 (1832/385)	2218 (1831/387)
Australian Terrier (CMO)	5,495,830	13,147	79 (73/6)	30 (24/6)	6527 (6012/515)	6511 (5875/636)
Basset Hound (CMO)	5,573,559	13,370	96 (87/9)	30 (28/2)	6535 (5917/618)	6709 (6073/636)
Cairn Terrier (CMO)	5,425,950	7373	55 (45/10)	17 (16/1)	3486 (3066/420)	3815 (3344/471)
Curly Coated Retriever (CMO)	5,326,349	4960	34 (34/0)	15 (15/0)	2405 (2248/157)	2506 (2260/246)
German Wirehaired Pointer (CMO)	5,519,945	11,713	63 (52/11)	28 (25/3)	5706 (5137/569)	5916 (5291/625)
Old English Sheepdog (CMO)	5,283,629	10,711	59 (52/7)	22 (17/5)	4966 (4175/791)	5664 (4558/1106)
Weimaraner (CMO)	5,201,857	8056	54 (48/6)	26 (21/5)	3859 (3183/676)	4117 (3307/810)

## Supplementary Materials

The following are available online at https://www.mdpi.com/2073-4425/11/2/163/s1: Table S1: Detailed information of all whole-genome sequences. Table S2: List of private variants of the sequenced CMO-affected Australian Terrier. Table S3. List of private variants of the sequenced CHS-affected American Staffordshire Terrier. Table S4. List of private variants of the sequenced CMO-affected Basset Hound. Table S5. List of private variants of the sequenced CMO-affected Cairn Terrier. Table S6. List of private variants of the sequenced CMO-affected Curly Coated Retriever. Table S7. List of private variants of the sequenced CMO-affected German Wirehaired Pointer. Table S8. List of private variants of the sequenced CMO-affected Old English Sheepdog. Table S9. List of private variants of the sequenced CMO-affected Weimaraner.

## References

[1] Thornburg L.P. (1979). Infantile cortical hyperostosis (Caffey-Silverman syndrome). Animal model: Craniomandibular osteopathy in the canine. Am. J. Pathol..

[2] Kamoun-Goldrat A., Le Merrer M. (2008). Infantile Cortical Hyperostosis (Caffey Disease): A Review. J. Oral Maxillofac. Surg..

[3] Gensure R.C., Cole W.G., Jüppner H., Gensure R.C., Mäkitie O., Barclay C., Chan C., Depalma S.R., Bastepe M., Abuzahra H. (2005). A novel COL1A1 mutation in infantile cortical hyperostosis (Caffey disease) expands the spectrum of collagen-related disorders. J. Clin. Investig..

[4] Kamoun-Goldrat A., Martinovic J., Saada J., Sonigo-Cohen P., Razavi F., Munnich A., Le Merrer M. (2008). Prenatal Cortical Hyperostosis With COL1A1 Gene Mutation. Am. J. Med. Genet..

[5] Kitaoka T., Miyoshi Y., Namba N., Miura K., Jüppner H., Kubota T., Ohata Y., Fujiwara M., Takagi M., Hasegawa T. (2014). Two Japanese familial cases of Caffey disease with and without the common COL1A1 mutation and normal bone density, and review of the literature. Eur. J. Pediatr..

[6] Merdler-rabinowicz R., Grinberg A., Jacobson J.M., Somekh I., Klein C., Lev A., Ihsan S., Habib A., Somech R., Simon A.J. (2019). Fetuin-A deficiency is associated with infantile cortical hyperostosis (Caffey disease). Pediatr. Res..

[7] Newton C.D., Nunamaker D.M. (1985). Textbook of Small Animal Orthopaedics.

[8] Alexander J.W. (1983). Selected Skeletal Dysplasias: Craniomandibular Osteopathy, Multiple Cartilaginous Exostoses, and Hypertrophic Osteodystrophy. Vet. Clin. North Am. Small Anim. Pract..

[9] Pastor K.F., Boulay J.P., Schelling S.H., Carpenter J.L. (2000). Idiopathic hyperostosis of the calvaria in five young bullmastiffs. J. Am. Anim. Hosp. Assoc..

[10] Padgett G.A., Mostosky U.V. (1986). Animal Model: The Mode of Inheritance of Craniomandibular Osteopathy in West Highland White Terrier Dogs. Am. J. Med. Genet..

[11] Hytönen M.K., Arumilli M., Lappalainen A.K., Owczarek-Lipska M., Jagannathan V., Hundi S., Salmela E., Venta P., Sarkiala E., Jokinen T. (2016). Molecular Characterization of Three Canine Models of Human Rare Bone Diseases: Caffey, van den Ende-Gupta, and Raine. PLoS Genet..

[12] Vagt J., Distl O. (2018). Complex segregation analysis of craniomandibular osteopathy in Deutsch Drahthaar dogs. Vet. J..

[13] Boycott K.M., Vanstone M.R., Bulman D.E., MacKenzie A.E. (2013). Rare-disease genetics in the era of next-generation sequencing: Discovery to translation. Nat. Rev. Genet..

[14] Ashley E.A. (2016). Towards precision medicine. Nat. Rev. Genet..

[15] Jagannathan V., Drögemüller C., Leeb T. (2019). Dog Biomedical Variant Database Consortium (DBVDC) A comprehensive biomedical variant catalogue based on whole genome sequences of 582 dogs and 8 wolves. Anim. Genet..

[16] Bauer A., Waluk D.P., Galichet A., Timm K., Jagannathan V., Sayar B.S., Wiener D.J., Dietschi E., Muller E.J., Roosje P. (2017). A de novo variant in the *ASPRV1* gene in a dog with ichthyosis. PLoS Genet..

[17] Hadji Rasouliha S., Bauer A., Dettwiler M., Welle M.M., Leeb T. (2018). A frameshift variant in the *EDA* gene in Dachshunds with X-linked hypohidrotic ectodermal dysplasia. Anim. Genet..

[18] Caduff M., Bauer A., Jagannathan V., Leeb T. (2017). A single base deletion in the *SLC45A2* gene in a Bullmastiff with oculocutaneous albinism. Anim. Genet..

[19] Spycher M., Bauer A., Jagannathan V., Frizzi M., De Lucia M., Leeb T. (2018). A frameshift variant in the COL5A1 gene in a cat with Ehlers-Danlos syndrome. Anim. Genet..

[20] Bridavsky M., Kuhl H., Woodruff A., Kornak U., Timmermann B., Mages N., Lupianez D.G., Symmons O., Ibrahim D.M., 99 Lives Consortium (2019). Crowdfunded whole-genome sequencing of the celebrity cat Lil BUB identifies causal mutations for her osteopetrosis and polydactyly. bioRxiv.

[21] Mauler D.A., Gandolfi B., Reinero C.R., Spooner J.L., Lyons L.A., 99 Lives Consortium (2017). Precision Medicine in Cats: Novel Niemann-Pick Type C1 Diagnosed by Whole-Genome Sequencing. J. Vet. Intern. Med..

[22] den Dunnen J.T., Dalgleish R., Maglott D.R., Hart R.K., Greenblatt M.S., McGowan-Jordan J., Roux A.-F., Smith T., Antonarakis S.E., Taschner P.E.M. (2016). HGVS Recommendations for the Description of Sequence Variants: 2016 Update. Hum. Mutat..

[23] Thorvaldsdóttir H., Robinson J.T., Mesirov J.P. (2013). Integrative Genomics Viewer (IGV): High-performance genomics data visualization and exploration. Brief. Bioinform..

[24] Karczewski K.J., Francioli L.C., Tiao G., Cummings B.B., Alföldi J., Wang Q., Collins R.L., Laricchia K.M., Ganna A., Birnbaum D.P. (2019). Variation across 141,456 human exomes and genomes reveals the spectrum of loss-of-function intolerance across human protein-coding genes. bioRxiv.

[25] Bendl J., Stourac J., Salanda O., Pavelka A., Wieben E.D., Zendulka J., Brezovsky J., Damborsky J. (2014). PredictSNP: Robust and Accurate Consensus Classifier for Prediction of Disease-Related Mutations. PLOS Comput. Biol..

[26] Choi Y., Chan A.P. (2015). PROVEAN web server: A tool to predict the functional effect of amino acid substitutions and indels. Bioinformatics.

[27] Sheth N., Roca X., Hastings M.L., Roeder T., Krainer A.R., Sachidanandam R. (2006). Comprehensive splice-site analysis using comparative genomics. Nucleic Acids Res..

[28] Nistala H., Mäkitie O., Jüppner H. (2014). Caffey disease: New perspectives on old questions. Bone.

[29] Campbell B.G., Wootton J.A.M., MacLeod J.N., Minor R.R. (2000). Sequence of Normal Canine COL1A1 cDNA 1 and Identification of a Heterozygous alpha1 (I) Collagen Gly208Ala Mutation in a Severe Case of Canine Osteogenesis Imperfecta. Arch. Biochem. Biophys..

[30] Ha B.G., Hong J.M., Park J.-Y., Ha M.-H., Kim T.-H., Cho J.-Y., Ryoo H.-M., Choi J.-Y., Shin H.-I., Chun S.Y. (2008). Proteomic profile of osteoclast membrane proteins: Identification of Na+/H+ exchanger domain containing 2 and its role in osteoclast fusion. Proteomics.

[31] Reese M.G., Eeckman F.H., Kulp D., Haussler D. (1997). Improved Splice Site Detection in Genie. J. Comput. Biol..

[32] Hiraoka S., Furuichi T., Nishimura G., Shibata S., Yanagishita M., Rimoin D.L., Superti-Furga A., Nikkels P.G., Ogawa M., Katsuyama K. (2007). Nucleotide-sugar transporter SLC35D1 is critical to chondroitin sulfate synthesis in cartilage and skeletal development in mouse and human. Nat. Med..

[33] Sugahara K., Kitagawa H. (2000). Recent advances in the study of the biosynthesis and functions of sulfated glycosaminoglycans. Curr. Opin. Struct. Biol..

